# Climate and Health Capacity Building for Health Professionals in Europe: A Pilot Course

**DOI:** 10.3389/ijph.2025.1608469

**Published:** 2025-06-18

**Authors:** Tara T. Chen, Ana-Catarina Pinho-Gomes, Nicola Hamacher, Marie Nabbe, Kirsten Duggan, Doris Zjalic, Danielly Magalhaes, Haley Campbell, Chiara Cadeddu, Christiana A. Demetriou, Souzana Achilleos, Ianis Delpla, Laurent Chambaud, Lore Leighton, Robert Otok, Kristie Hadley, Cecilia Sorensen

**Affiliations:** ^1^ Association of Schools of Public Health in the European Region (ASPHER), Brussels, Belgium; ^2^ Department of Geography and Environmental Management, University of Waterloo, Waterloo, ON, Canada; ^3^ Institute for Global Health, Faculty of Population Health Sciences, University College London, London, United Kingdom; ^4^ Global Consortium of Climate Change and Health Education, Mailman School of Public Health, Columbia University, New York, NY, United States; ^5^ HOPE European Hospital and Healthcare Federation, Brussels, Belgium; ^6^ Department of Life Sciences and Public Health, Catholic University of the Sacred Heart, Rome, Lazio, Italy; ^7^ School of Health Policy & Management, Erasmus University Rotterdam, Rotterdam, Netherlands; ^8^ Department of Primary Care and Population Health, School of Medicine, University of Nicosia, Nicosia, Cyprus; ^9^ École des Hautes Etudes en Santé Publique, Rennes, Brittany, France

**Keywords:** climate change, climate health, public health education, climate health literacy, competencies

## Abstract

**Objectives:**

The European Climate and Health Responder Course aimed to enhance health professionals’ knowledge, confidence, and preparedness to address climate-related health challenges.

**Methods:**

The course was delivered as a synchronous, online program targeting health professionals across diverse fields. Data on participant demographics, engagement, and knowledge improvement were collected through pre- and post-course surveys and course completion metrics. Statistical analysis measured changes in participants’ confidence and preparedness across targeted outcomes.

**Results:**

Of the 4,407 individuals who registered for the course, 21% completed the course, with the majority of them being from Europe and from academic and research institutions. The longitudinal survey revealed significant improvements in participants’ self-perceived outcomes across the three target domains from pre-course levels: communication, professional applicability, and self-efficacy.

**Conclusion:**

The pilot European Climate and Health Responder course highlights both the strong demand for and the effectiveness of climate change and health education for health professionals. The global interest further highlights the need for expanded climate-health education beyond the European Region.

## Introduction

Climate change is widely acknowledged as a significant public health threat [1]. Across Europe, record-breaking temperatures and accelerating environmental changes are intensifying the negative health impacts of climate change [2]. Europe has also witnessed several devastating disasters, such as 2023 heat waves that lead to approximately 47, 690 heat-related deaths [3], and the recent 2024 Valencia floods in Spain that triggered over 220 deaths [4]. These rapid environmental changes are exacerbating inequalities, disrupting access to healthcare, and impacting economic welfare, environmental justice, and other critical social determinants of health. In addition, climate change impacts exacerbate existing health disparities, and pose significant health threats on a population-wide scale [2, 5, 6]. The severity of climate-related health risks is highly dependent on the capacity of health systems to build climate resilience and protect populations. A critical step in achieving this is equipping health professionals with the knowledge and climate-health literacy skills needed to recognize, prepare for, and respond effectively to current and future climate-related threats [7]. However, in many cases, climate change education has lagged with rapidly emerging health risks and challenges posed by climate change, leaving many health professionals underprepared to address these pressing issues [8].

Although climate change has well-documented impacts globally, various social, economic, environmental, and cultural factors significantly influence climate action [9] highlighting the need for education tailored to specific geographic contexts. Health professionals across Europe - spanning public health professionals, physicians, nurses, allied health practitioners, local, regional, and national public health officials, hospital administrators, health system leaders, health educators and students, policymakers, environmental health specialists, and others working in climate-affected sectors - require comprehensive, action-oriented, interdisciplinary education [8]. They must be able to critically evaluate new knowledge and independently make complex decisions, from individual health appointments to community-wide adaptation planning. Given the complexity of the environmental crisis, a transdisciplinary approach is essential, drawing on the core concepts of One Health, Planetary Health, and EcoHealth, underscoring the need for collaboration across disciplines [10]. Each health profession brings unique expertise, enabling a comprehensive response to climate-related health challenges, which can better address the multifaceted impacts of climate change.

Current educational efforts in Europe to build capacity among health professionals in addressing climate-related health challenges are steadily growing but remain insufficient in several key areas [8, 11]. Programs such as the European Climate and Health Observatory offer valuable resources and frameworks to enhance knowledge and skills [12]. Additionally, some universities have begun integrating climate-health education into their program, particularly in medical education [13, 14]. However, significant gaps remain as training programs are not mandatory and there is a lack of faculty expertise [15] leaving substantial variability in climate-health literacy levels among professionals. In 2021, the Association of Schools of Public Health in the European Region (ASPHER) took an important step toward addressing the gap in climate and health education by releasing the climate change and health competencies for European public health professionals [16]. Building on this effort, ASPHER further emphasized the need for climate and health training with its 2022 Joint Statement “Moving towards the right to ‘health for all’ by training the public health and wider health workforce on climate change and health” which was presented as a call to action at the European Commission, through the EU Health Policy Platform [11]. In 2023, ASPHER’s statement, “A New Public Health Curriculum for a “New Normal” continued to highlight the critical role of health professionals in responding to the climate crisis, stressing the urgency for tailored curricula and capacity building across the public health workforce [12]. These efforts underscore the growing recognition of the need for comprehensive climate health education in the European public health sector [8].

In 2024, to address a localized focus of the training to the diverse sociopolitical and environmental contexts across Europe, the European Climate and Health Responder course was developed. This course was a collaboration between the Global Consortium on Climate and Health Education (GCCHE) at Columbia University and ASPHER. The course aimed to increase knowledge of climate and health, skills, communication, and action that reflect the crucial emerging roles of health professionals within Europe. Specifically, the goals of this course were to: (1) raise awareness of the impact of climate change on health and health practice and the professional responsibility to act (2) increase health professional communication about climate and health, (3) equip health professionals with knowledge and skills assist communities in adapting to climate change, (4) equip health professionals with knowledge and skills to accelerate health system decarbonization and resilience-building. This paper will describe the development of the European Climate and Health Responder Course and assess its effectiveness in meeting the stated goals. For that, a longitudinal survey was conducted with the course participants to determine whether the 15-hour live-virtual course effectively enhanced health professionals’ capacity to address climate and health threats.

## Methods

### Program Structure

The curricular foundation of this educational initiative followed both GCCHE and ASPHER core competencies for health professionals, a set of peer-reviewed global educational standards which cover the skills, knowledge and professional capabilities (including in policy, clinical medicine and public health) that all health professionals should have in the context of a changing climate [17]. From this foundation, ten discrete modules were developed, each focused on a specific emerging climate and health issue and with concrete learning objectives (see Supplementary Appendix Table SA1). This framework has previously been used for courses in other regional contexts to approach the perceived complexity that public health professionals often struggle with to translate climate-health knowledge into actionable interventions [18, 19]. For this course, this framework was adapted by an organizing committee of regional experts to reflect the specific challenges of the European region and to be specifically relevant to European healthcare professionals. Module classes were given on a weekly basis in 90-min live-virtual sessions from February 6 to April 9, 2024. All lectures were delivered by climate and health experts from the European region and were accompanied by online readings and/or other learning resources to encourage personal or regional application to demonstrate the direct relevance to their practice. Prior to the course start date, lecturers received a 1 h orientation on the overall course curriculum and the expectations of course participants. Participants who attended seven or more of the didactic training sessions and passed a final exam (with a score of 70% or greater) received a Climate and Health Responder Certificate of Participation, co-sponsored by ASHPER and the GCCHE. All aspects of the course were provided free of charge to participants. All sessions were recorded and made available online to participants and to the broader public.

### Audience and Recruitment

This educational initiative aimed to engage health professionals, broadly defined, and including public health and clinical professionals, hospital and health system leaders, policymakers, patient and community advocates, as well as those otherwise working to improve people’s health and wellbeing. The course recruitment commenced on December 19, 2023, with registration remaining open throughout its entire duration. Outreach to members of these groups, with invitations for course enrollment, was conducted via email and social media, utilizing the extended networks of ASPHER, the GCCHE, and members of the organizing committee.

### Enrollment and Participation

All participants who registered for the course completed a survey including demographic information (country of residence, occupation, place of work) and pre-course longitudinal questions (further detailed below). All registrants who attended at least seven sessions were sent an email on the last day of the course to take the final exam as well as the post-course longitudinal survey questions. Session attendance was tracked using participant emails connected to Zoom accounts and entered during registration and the final exam. Response data was collected in Qualtrics and was anonymized before being analyzed. The study protocol was approved by the Columbia University Institutional Review Board (AAAR4912).

### Survey Description

The longitudinal survey was structured to assess the effectiveness of this training course in three specific domains: Communication, Professional Applicability, and participants’ self-efficacy.

To evaluate communication skills, participants were asked to which degree they felt confident communicating about climate and health to colleagues, members of the community and, if applicable, patients. A 1-10 sliding scale was presented ranging from very low confidence (1) to high confidence (10).

To evaluate the professional applicability of the knowledge, participants were asked to which extent they: 1) believed the impacts of climate change on health affect their professional practice, 2) the degree to which they feel a sense of professional responsibility to help communities adapt to health threats and 3) the degree to which they feel a professional responsibility to help decarbonize and increase the resilience of the health system. A 1-10 sliding scale was presented ranging from “Not relevant” or “I feel no responsibility” (1) to “To a large extent” or “I feel a very high sense of responsibility” (10).

To evaluate participants’ self-efficacy, participants were asked to which degree they felt prepared 1) to help communities adapt to the health threats of climate change, and 2) to work towards health system decarbonization and resilience. A 1-10 sliding scale was presented ranging from “I feel unprepared” (1) to “I feel very prepared” (10).

### Analysis

All data from registration, course participation, and the final exam was organized and analyzed using R Studio and Microsoft Excel. Participants who completed both the registration (including pre-course survey questions) and the final exam (including post-course survey questions) were included in the analysis. For the longitudinal questions, the percent change was calculated for each multiple-choice response. This was listed as the delta “Change” and represents the impact of the course on participant responses. A Wilcoxon Paired Rank-Sum test was used to test the statistical strength of the results of the paired longitudinal data. The analysis did not take into account the number of sessions participants had participated in, outside of attending at least seven. Differences between group representation in the Registration and Survey Participant groupings were calculated in order to demonstrate the differences in course completion among various groups.

## Results

### Demographics

The course attracted 4,407 registrants from 165 countries, including participants from 30 European countries (Tables 1, 2). The highest representation came from Italy (700 registrants, 15.9%), the United Kingdom (334 registrants, 7.6%)), and France (186 registrants, 4.2%) (Table 1). Most participants (1979 individuals, 44.9%) were from the academic and research sector (Table 2). Nearly half (2010, 45.6%) were current students. The vast majority of participants, regardless of employment setting or student status, reported no prior training in climate and health (3142, 71.3%).

**TABLE 1:** Geographic distribution of registrants across the 30 most represented countries (Global, 2024).

#	Country	Number of registrants	Percent of total registrants	Number of participants who completed course, final exam and survey	Percent of total participants who completed course, final exam and survey	∆ (registration to survey participation)
1	Italy	700	15.9%	281	30.0%	+14.1%
2	United Kingdom	334	7.6%	81	8.7%	+1.1%
3	Germany	174	3.9%	54	5.8%	+1.8%
4	France	186	4.2%	33	3.5%	−0.7%
5	Portugal	87	2.0%	26	2.8%	+0.8%
6	Philippines^a^	157	3.6%	25	2.7%	−0.9%
7	Belgium	91	2.1%	19	2.0%	0.0%
8	Ireland	68	1.5%	19	2.0%	+0.5%
9	United States^a^	169	3.8%	18	1.9%	−1.9%
10	Switzerland	67	1.5%	17	1.8%	+0.3%
11	Nigeria^a^	165	3.7%	16	1.7%	−2.0%
12	India^a^	69	1.6%	15	1.6%	0.0%
13	Netherlands	64	1.5%	15	1.6%	+0.2%
14	Spain	78	1.8%	14	1.5%	−0.3%
15	Kazakhstan^a^	172	3.9%	11	1.2%	−2.7%
16	Kenya^a^	97	2.2%	11	1.2%	−1.0%
17	Turkey^a^	85	1.9%	11	1.2%	−0.8%
18	Austria	25	0.6%	10	1.1%	+0.5%
19	Canada^a^	67	1.5%	10	1.1%	−0.5%
20	Greece	22	0.5%	10	1.1%	+0.6%
21	Lithuania	23	0.5%	10	1.1%	+0.5%
22	Romania	16	0.4%	9	1.0%	+0.6%
23	Malaysia^a^	36	0.8%	8	0.9%	0.0%
24	Peru^a^	95	2.2%	8	0.9%	−1.3%
25	Sweden	21	0.5%	8	0.9%	+0.4%
26	Jamaica^a^	61	1.4%	7	0.7%	−0.6%
27	Pakistan^a^	37	0.8%	7	0.7%	−0.1%
28	Bangladesh^a^	61	1.4%	6	0.6%	−0.7%
29	Ecuador^a^	48	1.1%	6	0.6%	−0.4%
30	Poland	21	0.5%	6	0.6%	+0.2%
	Europe total^a^	1977	45%	612	65.5%	
	Non-Europe total	1319	29.9%	159	17%	

^a^ Non-European countries.

**TABLE 2:** Affiliated organizations or sectors of course registrants and survey participants (N = 4,407 registrants, n = 936 participants) (Global, 2024).

Affiliated organizations or sectors	Number of registrants	Percent of total registrants	Number of participants who completed course, final exam and survey	Percent of total participants who completed course, final exam and survey	∆ (registration to survey participation)
Academic/Research Institution	1979	44.9%	506	54.1%	9.2%
Government/Intergovernmental	1097	24.9%	180	19.2%	−5.7%
Non-governmental organization/Non-profit	483	11.0%	65	6.9%	−4.0%
Other	527	12.0%	134	14.3%	2.4%
Private sector	321	7.3%	51	5.4%	−1.8%

### Course Participants and Completion

Of the 4,407 individuals who registered for the course, 936 (21%) completed the course by attending at least 7 sessions, passing the final exam, and completing the final survey. 612 (65.5%) of 936 participants who completed the course were from Europe (Table 1). 506 (54.1%) of 936 participants who completed the course were from academic and research institutions, and 180 (19.2%) of 936 participants were from the government sector (Table 2).

### Longitudinal Survey

The longitudinal survey revealed significant improvements in participants’ outcomes across three target domains from pre-course levels: communication, professional applicability, and preparation and engagement.

Table 3 illustrates the impact of the course on participants’ confidence in communicating the health impacts of climate change across three distinct contexts: colleagues, community members, and patients. Participants reported significant improvements, with confidence response levels increasing by 31.1% (p < 0.01) when engaging with colleagues, 31.7% (p < 0.01) with community members, and 29.6% (p < 0.01) with patients.

**TABLE 3:** Mean response levels of participants regarding communication skills as measured in the longitudinal survey, with the average change observed before and after the course (Global, 2024).

Questions	n	Pre (x̄)	Post (x̄)	Mean ∆	Mean ∆ (%)	p-value
1. How confident do you feel communicating with WORK COLLEAGUES about the health impacts of climate change?	932	6.01	7.88	+1.87	+31.1	<0.01
2. How confident do you feel communicating with MEMBERS OF YOUR COMMUNITY about the health impacts of climate change?	932	5.96	7.85	+1.89	+31.7	<0.01
3. IF APPLICABLE: How confident do you feel communicating with PATIENTS about the health impacts of climate change?	553	6.04	7.83	+1.79	+29.6	<0.01

Responses were given on a scale from 1-10, where 1 indicated “Not confident”, and 10 indicated “Very confident”.

Statistically significant improvements were seen in each of the eight longitudinal questions utilized. Of particular note were the improvements in preparation.

Participants showed significant increases in their response levels regarding the recognition of the health impacts of climate change in their professional practice and their sense of responsibility in addressing related health risks when comparing pre and post-course responses. There was a 26.8% increase in the response levels regarding the recognition of climate change’s relevance to professional work (p < 0.01), as well as a 17.1% increase in the response levels related to responsibility to help communities adapt to climate health threats (p < 0.01). Additionally, a 16.7% increase in response levels related to the responsibility to help their health systems decarbonize and become more resilient to the impacts of climate change (p < 0.01) (Table 4).

**TABLE 4:** Mean response levels of participants regarding professional applicability as measured in the longitudinal survey, with the average change observed before and after the course (Brussels, Belgium. 2024).

Questions	n	Pre (x̄)	Post (x̄)	Mean ∆	Mean ∆ (%)	p-value
1. To what extent do the impacts of climate change on health affect the work you do in your professional practice?	932	6.15	7.80	+1.65	+26.8	<0.01
2. To what degree do you feel a sense of professional responsibility to: Help your community adapt to the health threats of climate change?	936	7.21	8.44	+1.23	+17.1	<0.01
3. To what degree do you feel a sense of professional responsibility to: Help your health system decarbonize and become more resilient to the impacts of climate change?	936	7.13	8.32	+1.19	+16.7	<0.01

Responses to Question 1 were given on a scale from 1 to 10, where 1 indicated “Not relevant - Climate change does not impact my professional practice,” and 10 indicated “To a large extent - Climate change impacts all facets of my professional practice.” Responses to Questions 2 and 3 were also given on a scale from 1 to 10, where 1 indicated “I feel no responsibility,” and 10 indicated “I feel a very high sense of responsibility.”

The results indicate significant increases in participants’ response levels related to preparedness to address climate change-related health issues when comparing pre and post-course responses. *Participants reported a* 41.7% improvement in response level on preparedness to help their communities to adapt to climate health threats (p < 0.001) and 42.3% improvement in their response level on preparedness to assist to help their health systems decarbonize and become more resilient to the impacts of climate change (p < 0.01).) (Table 5).

**TABLE 5:** Mean confidence levels of participants regarding preparation and engagement as measured in the longitudinal survey, with the average change observed before and after the course (Global, 2024).

Questions	n	Pre (x̄)	Post (x̄)	Mean ∆	Mean ∆ (%)	p-value
1. To what degree do you feel prepared to: Help your community adapt to the health threats of climate change?	936	5.16	7.31	+2.15	+41.7	<0.01
2. To what degree do you feel prepared to: Help your health system decarbonize and become more resilient to the impacts of climate change?	936	4.94	7.03	+2.09	+42.3	<0.01

Responses were given on a scale from 1 to 10, where 1 indicated “I feel unprepared” and 10 indicated “I feel very prepared.”

## Discussion

The European Climate and Health Responder Course demonstrated both the need for and willingness of health professionals to engage in climate change and planetary health education. It also illustrated the effectiveness of such programming in making an impact on health professionals’ confidence and ability to competently engage with climate change in their professional capacities.

Tracking of participant demographics revealed widespread global engagement in this course with registrants from 165 countries–of which 103 countries had participants who completed the course. This level of engagement outside of Europe in a course specifically focused on the European region demonstrates the need for further health professional climate change and planetary health education globally. The courses’ diverse range of guest lecturers and case studies provided an introductory overview of the present landscape of the climate and health issues in Europe, as well as relevant, innovative case studies. As the course was organised by the GCCHE and the Climate and Health Working Group under ASPHER, the goal of this study was to address the urgent need for an introductory course. While other environmental changes closely intertwined with climate change, the pedagogical decision to focus on climate-health links was guided by the urgent need to provide the building blocks in this evolving area. The focus of each session was intentionally designed to be well-rounded, noting important emerging topics in climate health discussions, such as Session 8: Climate Litigation and Health, and Session 9: Communication, Engagement, and Advocacy in Climate Change. This knowledge provision is crucial for providing the tools to public health professionals to translate complex climate science into actionable public health messages, as well as gain insights into how legal avenues can drive policy changes. Future iterations of the course may explore on an expanded, systems-based approach to planetary health.

Additional enabling factors for the success of the course may be attributed to ASPHER’s previous work in climate and health which has established credibility and influence. As ASPHER is part of a wide array of climate and health partnerships, networks and working groups in Europe, it was a natural fit for the collaboration with the GCCHE. This initiative has enriched the curriculum and strengthened opportunities for practical application and connection among the partners and its participants. While individual motivation plays a key role in course participation, institutional support was critical for enabling sustained engagement with climate and health education. Many participants accessed the course outside of their regular working hours, highlighting the importance of institutional leadership to invest in the climate-health literacy of their workforce. Moreover, the high registration was enabled by the technical flexibility of participating virtually with no cost and the attractiveness of receiving a certificate of completion, providing credibility for students and professionals. There was a high percentage of participants based in academic institutions (54%) and those who identified as students (45.6%) also highlights the interest in climate change and health education by health professional faculty as well as the students they serve. This was attributed to ASPHER’s network of Schools of Public Health, allowing early marketing to encourage faculty to embed the course into their existing curriculum, incentivizing students to participate.

### Limitations

While the course yielded overall positive outcomes, some challenges and limitations are worth highlighting. While the evaluation suggests positive shifts in communication confidence, professional applicability, and self-efficacy, the absence of a control group limits the ability to attribute these changes solely to the course rather than to external influences. The longitudinal survey is based on self-reported data thus responses may be subject to social desirability bias, potentially inflating perceived improvements which does not include willingness to act or self-reported actions as well. Furthermore, although broad institutional categories were recorded, more granular data on participants’ specific health professions were not systematically collected. Future iterations of the evaluation will aim to capture this information to better contextualize outcomes across different potential stages.

Challenges related to the overall competition rate may have been due to a multitude of factors including the difficulties brought regarding teaching a live synchronous online course across multiple time zones, access to internet and interest in the topic. Given the variability of professional backgrounds of registrants, there are challenges to find a suitable time that works for all participants. Live sessions were recorded, and the content was available on the course registration website for free afterwards. Participants had the flexibility to engage the material at their own pace. However, due to technical constraints, this could not be counted as attendance for the certificate, which may have led to an incomplete picture of course completion. Furthermore, some participants may not have been accounted for due to the use of multiple emails. Efforts were made to mitigate this by informing participants in advance and reminding them of email tracking throughout the course. Additionally, differences in country-level registration and participation as a proportion are shown in Table 1 and demonstrate large changes among some countries indicating a desire to engage, but potential difficulties in doing so. This may be due to competing work priorities due to the current array of public health challenges. Moreover, institutional priorities do not always align with climate and health education, thus participation may be limited. Further exploration of reasons underpinning variable engagement would be of interest in future courses.

### Conclusion

As climate change continues to accelerate, the role of health professionals as leaders within these domains will grow, further highlighting the necessity and impact of this type of education. The pilot European Climate and Health Responder course highlights both the strong demand for and the effectiveness of climate change and health education for health professionals. The program not only enhanced participants’ confidence and competencies in addressing climate-related health challenges, but also demonstrated the global appeal of such initiatives, with engagement from registrants in 165 countries. This global interest further highlights the need for expanded and more equitable accessible climate-health education beyond the European Region.

Future iterations of the course in Europe will build on these successes by placing a stronger emphasis on practical applications and specialized content tailored to specific professional roles in the health sector. Future focus aims to better equip health professionals to implement climate adaptation and mitigation strategies within their unique contexts, such as sustainable health system planning. Additionally, targeted modules designed to address regional climate-health challenges and profession-specific needs will further enhance the relevance and impact of the course goals. By refining the course to provide more specialized, action-oriented education, future offerings will better prepare health professionals to lead in addressing the escalating health impacts of climate change across diverse settings.
