# Supplementary material for: Climate and Health Capacity Building for Health Professionals in Europe: A Pilot Course
Source: Int J Public Health. 2025 Jun 18;70:1608469. doi: 10.3389/ijph.2025.1608469 (PMC12216089; doi:10.3389/ijph.2025.1608469)
Supplement: Supplementary file 1 [file Supplementaryfile1.docx]

Table A1. Focus of Each Responder Course session (Global, 2024).

| **Date** | **Session Focus** |
| --- | --- |
| Session 1: February 6, 2024 | Climate Change and Health |
| Session 2: February 13, 2024 | Extreme Temperatures |
| Session 3: February 20, 2024 | Wildfires and Air Quality |
| Session 4: February 27, 2024 | Water Supply and Sanitation |
| Session 5: March 5,  2024 | Vector Borne/ Zoonoses Diseases |
| Session 6: March 12,  2024 | Pathways to a Healthy Net Zero Future |
| Session 7: March 19,  2024 | Sustainable Health Care |
| Session 8: March 26,  2024 | Climate Litigation and Health |
| Session 9: April 2,  2024 | Communication, Engagement and Advocacy in Climate Change |
| Session 10: April 9,  2024 | Europe in a Global Context: Climate Justice |

Table A2. Country of residence for all course registrants (registered for the course) and participants (completed the course) (Global, 2024).

| **Country Name** | **Regis-**  **tration** | **Partic-**  **ipants** | **Country Name** | **Regis-**  **tration** | **Partic-**  **ipants** | **Country Name** | **Regis-**  **tration** | **Partic-**  **ipants** |
| --- | --- | --- | --- | --- | --- | --- | --- | --- |
| Afghanistan | 1 | 0 | Gambia | 3 | 0 | Oman | 3 | 0 |
| Albania | 6 | 0 | Georgia | 14 | 3 | Pakistan | 37 | 7 |
| Algeria | 2 | 0 | Germany | 174 | 54 | Palestinian Territories | 1 | 0 |
| American Samoa | 1 | 0 | Ghana | 19 | 4 | Panama | 6 | 1 |
| Angola | 1 | 0 | Greece | 22 | 10 | Papua New Guinea | 3 | 0 |
| Antarctica | 1 | 0 | Grenada | 20 | 0 | Paraguay | 5 | 1 |
| Antigua and Barbuda | 18 | 4 | Guadeloupe | 1 | 0 | Peru | 95 | 8 |
| Argentina | 42 | 3 | Guatemala | 10 | 0 | Philippines | 157 | 25 |
| Armenia | 1 | 1 | Guinea | 1 | 0 | Poland | 21 | 6 |
| Australia | 8 | 1 | Guinea-  Bissau | 2 | 0 | Portugal | 87 | 26 |
| Austria | 25 | 10 | Guyana | 19 | 5 | Puerto Rico | 7 | 1 |
| Azerbaijan | 1 | 0 | Haiti | 19 | 5 | Réunion | 2 | 0 |
| Bahamas | 16 | 5 | Honduras | 3 | 0 | Romania | 16 | 9 |
| Bangladesh | 61 | 6 | Hong Kong SAR | 4 | 1 | Rwanda | 1 | 1 |
| Barbados | 5 | 0 | Hungary | 6 | 0 | Saint Kitts and Nevis | 3 | 0 |
| Belarus | 2 | 0 | Iceland | 1 | 0 | Saint Lucia | 9 | 1 |
| Belgium | 91 | 19 | India | 69 | 15 | Saint Vincent and the Grenadines | 5 | 1 |
| Belize | 7 | 0 | Indonesia | 17 | 5 | Saudi Arabia | 1 | 0 |
| Benin | 3 | 0 | Iraq | 1 | 0 | Senegal | 7 | 2 |
| Bolivia | 13 | 1 | Ireland | 68 | 19 | Serbia | 4 | 1 |
| Botswana | 1 | 0 | Israel | 32 | 5 | Singapore | 3 | 2 |
| Brazil | 45 | 3 | Italy | 700 | 281 | Sint-Maarten (Dutch) | 1 | 0 |
| Brunei Darussalam | 2 | 1 | Jamaica | 61 | 7 | Slovakia | 3 | 2 |
| Bulgaria | 5 | 1 | Japan | 6 | 0 | Somalia | 49 | 3 |
| Burkina Faso | 2 | 0 | Jordan | 2 | 0 | South Africa | 22 | 3 |
| Burundi | 2 | 0 | Kazakhstan | 172 | 11 | South Sudan | 1 | 0 |
| Cambodia | 6 | 2 | Kenya | 97 | 11 | Spain | 78 | 14 |
| Cameroon | 19 | 3 | Korea, Republic of | 5 | 0 | Sri Lanka | 4 | 0 |
| Canada | 67 | 10 | Kosovo | 3 | 1 | Sudan | 5 | 2 |
| Cayman Islands | 1 | 0 | Kyrgyzstan | 12 | 1 | Suriname | 11 | 3 |
| Chad | 1 | 0 | Latvia | 2 | 2 | Sweden | 21 | 8 |
| Chile | 19 | 1 | Lebanon | 2 | 1 | Switzerland | 67 | 17 |
| China | 1 | 1 | Liberia | 1 | 0 | Taiwan | 4 | 0 |
| Colombia | 37 | 4 | Lithuania | 23 | 10 | Tajikistan | 2 | 1 |
| Congo | 7 | 0 | Luxembourg | 2 | 0 | Tanzania | 8 | 3 |
| Congo, Democratic Republic of the | 11 | 2 | Madagascar | 1 | 1 | Thailand | 10 | 2 |
| Costa Rica | 9 | 1 | Malawi | 5 | 1 | Togo | 4 | 0 |
| Côte d'Ivoire | 1 | 0 | Malaysia | 36 | 8 | Trinidad and Tobago | 42 | 4 |
| Croatia | 2 | 0 | Mali | 1 | 0 | Tunisia | 3 | 1 |
| Curaçao | 2 | 0 | Malta | 8 | 5 | Turkey | 85 | 11 |
| Cyprus | 3 | 1 | Mauritius | 2 | 0 | Turks and Caicos Islands | 2 | 0 |
| Czech Republic | 8 | 0 | Mexico | 44 | 5 | Uganda | 28 | 5 |
| Denmark | 12 | 3 | Moldova, Republic of | 30 | 5 | Ukraine | 13 | 5 |
| Djibouti | 2 | 0 | Mongolia | 10 | 3 | United Arab Emirates | 15 | 3 |
| Dominica | 15 | 2 | Montenegro | 1 | 0 | United Kingdom | 334 | 81 |
| Dominican Republic | 2 | 0 | Morocco | 4 | 2 | United States | 169 | 18 |
| Ecuador | 48 | 6 | Mozambique | 6 | 1 | Uruguay | 11 | 2 |
| Egypt | 16 | 2 | Myanmar | 3 | 2 | Uzbekistan | 3 | 0 |
| El Salvador | 3 | 1 | Namibia | 1 | 0 | Venezuela | 13 | 1 |
| Estonia | 3 | 1 | Nepal | 16 | 2 | Vietnam | 3 | 0 |
| Ethiopia | 21 | 2 | Netherlands | 64 | 15 | Virgin Islands (British) | 1 | 0 |
| Fiji | 2 | 0 | Nicaragua | 3 | 0 | Virgin Islands (U.S.) | 1 | 0 |
| Finland | 25 | 2 | Nigeria | 165 | 16 | Yemen | 3 | 0 |
| France | 186 | 33 | North Macedonia | 3 | 1 | Zambia | 19 | 3 |
| French Guiana | 2 | 1 | Norway | 5 | 1 | Zimbabwe | 5 | 0 |
